# Knockout of Mucin 1 inhibits the proliferation, migration, and invasion of human MDA-MB-231 cells by blocking autophagy flow

**DOI:** 10.1590/1414-431X2026e15075

**Published:** 2026-04-27

**Authors:** Zhimei Huang, Jiayao Zhao, Qinqin Zhang, Yu Luo, Wenqing Chen, Zhengchun Liu, Xiuli Liu

**Affiliations:** 1 Department of Guangxi Clinical Medical Research Center for Neurological Diseases, The First Affiliated Hospital of Guilin Medical University, Guilin, Guangxi Zhuang Autonomous Region, China; 2 Department of Laboratory Medicine, Guilin People’s Hospital, Guilin, Guangxi Zhuang Autonomous Region, China; 3 Department of Thyroid and Breast Surgery, Nanxishan Hospital of Guangxi Zhuang Autonomous Region, Guilin, Guangxi Zhuang Autonomous Region, China; 4 Department of Radiotherapy, The First Affiliated Hospital of Guilin Medical University, Guilin, Guangxi Zhuang Autonomous Region, China; 5 Department of Oncology, The First Affiliated Hospital of Guilin Medical University, Guilin, Guangxi Zhuang Autonomous Region, China

**Keywords:** Triple negative breast cancer, MDA-MB-231 cells, MUC1, Autophagy flow, Lysosomal damage

## Abstract

To investigate the effects of Mucin 1 (MUC1) in human triple-negative breast cancer MDA-MB-231cells, the MDA-MB-231 cell line with MUC1 knockout (231-MUC1-KO) was constructed by CRISPR/Cas9 gene editing. Cell proliferation was evaluated using EDU and colony formation assays, and cell migration and invasion were detected by transwell assay. Autophagy flow was assessed by western blot and Ad-mCherry-GFP-LC3B dual-fluorescence system and validated by lysosome inhibitor barfimycin A1 and autophagy inducer rapamycin. Key proteins of autophagosomes and lysosomal fusion (STXl7, SNAP29) and lysosomal tagged protein (LAMP1) were detected by western blot, and lysosomal pH was evaluated by Lysotracker Red fluorescence. MUC1 expression was low in human normal breast epithelial cells MCF-10A, but was highly expressed in human MDA-MB-231 cells and tissues. Successful MUC1 knockout was confirmed by gene sequencing, RT-qPCR, and western blot. Loss of MUC1 gene expression in 231-MUC1-KO significantly reduced proliferation, migration, and invasion. Compared with the control group, MUC1 knockout led to a significant increase of autophagy-related proteins LC3-II and p62, which is consistent with the effect of lysosome inhibitor bleomycin A1. After adding the autophagy inducer rapamycin, compared with the control group, the accumulation of LC3-II and p62 proteins also further increased. The expression level of LAMP1 was downregulated and the lysosome pH increased, but the expression levels of STXl7 and SNAP29 were not affected. These findings suggest that MUC1 promotes malignant behaviors in MDA-MB-231 cells by regulating autophagic flow, likely through lysosomal dysfunction-mediated autophagy blockade.

## Introduction

The number of new breast cancer cases reached 2.3 million in 2022 in the world, ranking second in the number of new cancer cases (1). However, triple-negative breast cancer (TNBC), which does not express estrogen (ER), progesterone (PR), and human epidermal growth factor receptor 2/neu receptors (HER2), cannot benefit from endocrine therapy and targeted therapy, so it lacks effective treatment means, posing a serious threat to people’s life and health (2). Therefore, it is particularly urgent to find new therapeutic targets and strategies to improve the current treatment of TNBC.

Autophagy is a dynamic process that includes the initiation of autophagy, autophagosome formation, fusion of autophagosomes and lysosomes (3), and degradation of autolysosomal contents. This process is collectively known as “autophagic flow”. Obstacles in any link may lead to the failure of autophagy, referred to as “autophagic flow arrest”, which can ultimately affect cell proliferation and differentiation (4-8).

Mucin 1 (MUC1) is a transmembrane mucin that is aberrantly highly expressed in more than 90% of early TNBC lesions (9-11). We had previously found that MUC1 expression was associated with tumor size, histological grade, lymph node metastasis, pathological stage, and 5-year disease-free survival in breast cancer (12). Moreover, a growing number of studies have shown that targeting MUC1 can effectively inhibit the proliferation and promote apoptosis of TNBC cells (13-17). Therefore, MUC1 is an ideal target for exploring effective therapies for TNBC. At present, the relationship between MUC1 and autophagic flow is not very clear. Hiraki et al. (18) found that MUC1-C activated autophagy in TNBC cells (MDA-MB-468) through the MEK-ERK and PI3K-Akt pathways. MUC1 interacts with EGFR, thereby regulating the autophagy pathway EGFR-PI3K-Akt-mTOR (19-22). Therefore, it is possible that MUC1 plays an important role in autophagic flow regulation in TNBC.

In this study, CRISPR/Cas9, which enables precise and efficient gene editing, was used to knock down MUC1 expression in MDA-MB-231 cells. The effects of MUC1 knockout on the proliferation, migration, and invasion of MDA-MB-231 cells were then examined, along with its impact on autophagy flow and the underlying mechanism.

## Material and Methods

### Bioinformatics analysis

UALCAN (http://ualcan.path.uab.edu/index.html) is a web tool used to analyze tumor transcriptome data. It provides publicly accessible cancer transcriptome data (TCGA mRNA sequencing), with graphs and plots of gene expression. MUC1 mRNA expression in adjacent normal tissue and cancerous breast tissue specimens and the difference in subtypes were compared using the UALCAN web tool. The Human Protein Atlas (HPA) database (https://www.proteinatlas.org/) was used to evaluate the staining patterns of MUC1 in different breast tumor tissues.

### Cell culture

The human triple-negative breast cancer cell line MDA-MB-231 and the normal human breast epithelial cells MCF10A were obtained from the Procell Biotechnology Company (China). DMEM high glucose medium, 0.25% trypsin solution (including EDTA), penicillin/streptomycin solution, and fetal bovine serum (FBS) were purchased from Gibco (Invitrogen, USA). MDA-MB-231 was cultured at 37°C, containing 5% CO_2_ in a humidified incubator. All experiments were performed while the cells were in the log growth phase.

### Immunofluorescence

MDA-MB-231 cells were seeded onto coverslips and the cells were fixed with 4% paraformaldehyde for 15 min at room temperature. After washing three times in phosphate buffered saline (PBS), the cells were permeabilized with 0.1%Triton X-100/PBS for 10 min. Then, after washing with PBS, the cells were blocked with 10% BSA for 30 min at room temperature. Then, the primary antibody MUC1 was incubated for 2 h at room temperature (Abcam, UK, ab109185, 1:500 dilution). Subsequently, they were incubated at room temperature for 30 min using the Alexa Flour@488-labeled secondary antibody (Abcam, ab150077, 1:1000 dilution), after which the microscope slides were sealed with anti-fluorescent quenching agent (DAPI, Solarbio, China). Finally, images were taken using a laser confocal microscope (Nikon, Japan, 20×).

### Construction of the MUC1 gene knockout cell lines

Using the principle of CRISPR/Cas9 gene editing technology, targets for cellular MDA-MB-231 and MUC1-255 transcripts were designed at the upstream and downstream of exon 2 and exon 8 (guide RNA with less off-target was selected as the target). The sequence for gRNA-A1 was ACCCTGAGAGTGGGTACCAG GGG and for gRNA-A2, AAGTGTCCGAGAAATTGGTGGGG. Target cells with good log phase growth were digested and centrifuged (180 *g* at 25°C for 4 min) to remove the supernatant, and were resuspended using PBS. Cells (1×10^6^) were placed in an Eppendorf (EP) tube, and the cell precipitate was obtained by centrifugation (180 *g* at 25°C for 4 min). gRNA and Cas9 proteins were combined into RNP complexes, which were used to resuspend the cells. The target gene MUC1 of the target cells was knocked down by Invitrogen Neon™ transfectant (Thermo Fisher Scientific, USA, Cat #MPK5000), and then individual cells were isolated for monoclonization and verified by PCR and sequencing. The positive monoclone (231-MUC1-KO) was obtained by amplification and culture.

### Reverse transcription-quantitative PCR (RT-qPCR)

Total RNA from 231-MUC1-KO cells was extracted using TRIzol^®^ reagent (Invitrogen). Reverse transcription of the extracted total RNA using a quantitative PCR reverse transcription kit (TaKaRa, Japan, Code No. RR047A) was performed to obtain each set of cDNA. Then, using the cDNA as a template, the MUC1 expression levels were determined by the Applied Biosystems™ Power SYBR™ Green PCR Mix (Invitrogen, UK, REF4367659) and LightCycler 96 fluorescence quantitative PCR instrument (Roche, Germany). The specific primer sequences of the target genes were as follows: β-actin (PF5' to 3': GGCACCCAGCACAATGAAG; PR5' to 3': TCCTGCTTGCTGATCCACAT), MUC1 (PF5' to 3': TGCTGCTCCTCACAGTGCTTAC; PR5' to 3': AGTAGTCGGTGCTGGGATCTTC). The primers were designed and synthesized by the Biotech Shanghai Company, China. The relative mRNA expression levels of target genes were normalized to those of β-actin using the 2−ΔΔCq method.

### Western blotting analysis

231-MUC1-KO cells were collected in 1.5 mL EP tubes and samples were lysed on ice using RIPA lysis buffer for total protein extraction (Beyotime, China). Protein concentrations were also measured using the BCA method (Beyotime). The supernatant was transferred to a PVDF membrane by SDS polyacrylamide gel (5% concentrate and 12.5% separation). PVDF membranes were blocked in 5% skim milk for 60 min at room temperature and incubated with the following specific antibodies overnight (16-18 h) at 4°C: MUC1 (Abcam, ab109185, 1:1000 dilution), LC3B (Abcam, ab192890,1:1000 dilution), P62 (Abcam, ab109012, 1:1000 dilution), SNAP29 (Proteintech, China, Cat No. 12704-1-AP, 1:1000 dilution), STX17 (Proteintech, Cat No. 17815-1-AP, 1:1000 dilution), and LAMP1 (Cell Signaling Technology, USA, #9091,1:1000 dilution). Membranes were washed in tris-buffered saline with Tween-20 (TBST) three times for 8 min each time. They were incubated with anti-rabbit/mouse fluorescently labeled IgG for 1 h at room temperature. Protein bands were detected after washing the membrane three times in TBST. Finally, the bands were analyzed using ImageJ software (NIH, USA) and the relative content of the target protein is reported as the gray value of the target protein/β-actin. The autophagy enhancer Rapamycin (10 nm concentration for 12 h) and the late autophagy inhibitor Bafilomycin A1 (50 nm concentration for 2 h) used in this study were purchased from MedChemExpress (USA).

### EDU cell proliferation assay

231-MUC1-KO cells were seeded at 2.5×10^4^/well onto 24-well plates and grown overnight. An equal volume of 37°C preheated 2× EdU solution (Beyotime) was added to the 24-well plate to change the final concentration of EdU. After further incubation of cells for 2 h, the medium was removed and 4% paraformaldehyde (Beyotime) was added for 15 min at room temperature. The detergent was washed three times and the permeabilized solution (PBS with 0.3% Triton X-100) was incubated for 10-15 min at room temperature. The cells were washed 1-2 times with washing solution, and 100 μL of EDU reaction mixture was added to each well and incubated at room temperature for 30 min without light. The cells were washed three times and the Hoechst 33342 solution (Beyotime, China) was used for nuclear staining for 15 min. EdU-positive cells were visualized and quantified under a fluorescence microscope (Olympus, Japan, 20×). By quantifying the relative intensity of fluorescent-positive cells, cell proliferation activity can be quantitatively evaluated.

### Plate clone formation assay

231-MUC1-KO cells were seeded onto 6-well plates at 5×10^2^/well, fixed with 4% paraformaldehyde for 15 min, and stained with 1% crystal violet for 30 min. Photographs were taken after washing with PBS. Cell colonies were counted using the ImageJ software (NIH, USA).

### Cell invasion and migration assay

231-MUC1-KO cells were homogeneously inoculated at 2×10^4^ cells per well into matrix gel-coated Transwell chambers (Corning, USA). Non-coated Transwell chambers were used for cell migration assays. The lower chamber contained 600-800 μL of fresh culture medium containing 20% FBS. After 48 h of incubation, the chamber was removed, the upper layer of the chamber was gently wiped off with a cotton swab, 4% paraformaldehyde (Beyotime) was added at room temperature for 15 min, and 0.25% crystal violet staining solution was added for 15 min. The number of cells was counted in five random visual fields under a microscope.

### Observation of autophagy flow by transfection with Ad-mCherry-GFP-LC3

231-MUC1-KO cells were treated at the density of 8×10^4^ cells/well and seeded onto laser confocal small dishes. Adenoviral infection was performed when the cell density was roughly 50-60% after 24 h. Adenoviruses were thawed on ice. After 24 h of adenovirus infection, the medium containing the virus solution was discarded and the cells were washed once with PBS buffer. Subsequently, fresh DMEM complete medium was added to continue the culture. The expression of intracellular fluorescence can be observed only 24-48 h after viral transfection using a laser confocal microscope (Nikon, 40×).

### Lysosomal acidic environment staining with Lyso-Tracker Red probe

231-MUC1-KO cells were seeded in laser confocal dishes and grown to appropriate concentrations. Cells were then removed from the cell culture medium, the Lyso-Tracker Red (Beyotime) was prepared and pre-incubated, and the cells were incubated for 37°C for 30 min. The Lyso-Tracker Red staining working solution was removed and fresh cell culture medium was added. Lysosomal acidity is usually observed and photographed with a laser confocal microscope (Nikon, 40×).

### Statistical analysis

All data presented in this study are representative of at least three independent replicates. Quantitative data are reported as means±SE and were analyzed using GraphPad Prism 9.5.1 software (GraphPad Software Inc., USA). Differences between the two groups were compared using unpaired Student's *t*-test for 2 groups and ANOVA for three or more groups. P<0.05 was considered to indicate a statistically significant difference.

## Results

### MUC1 upregulation in human breast cancer

**Figure 1:**
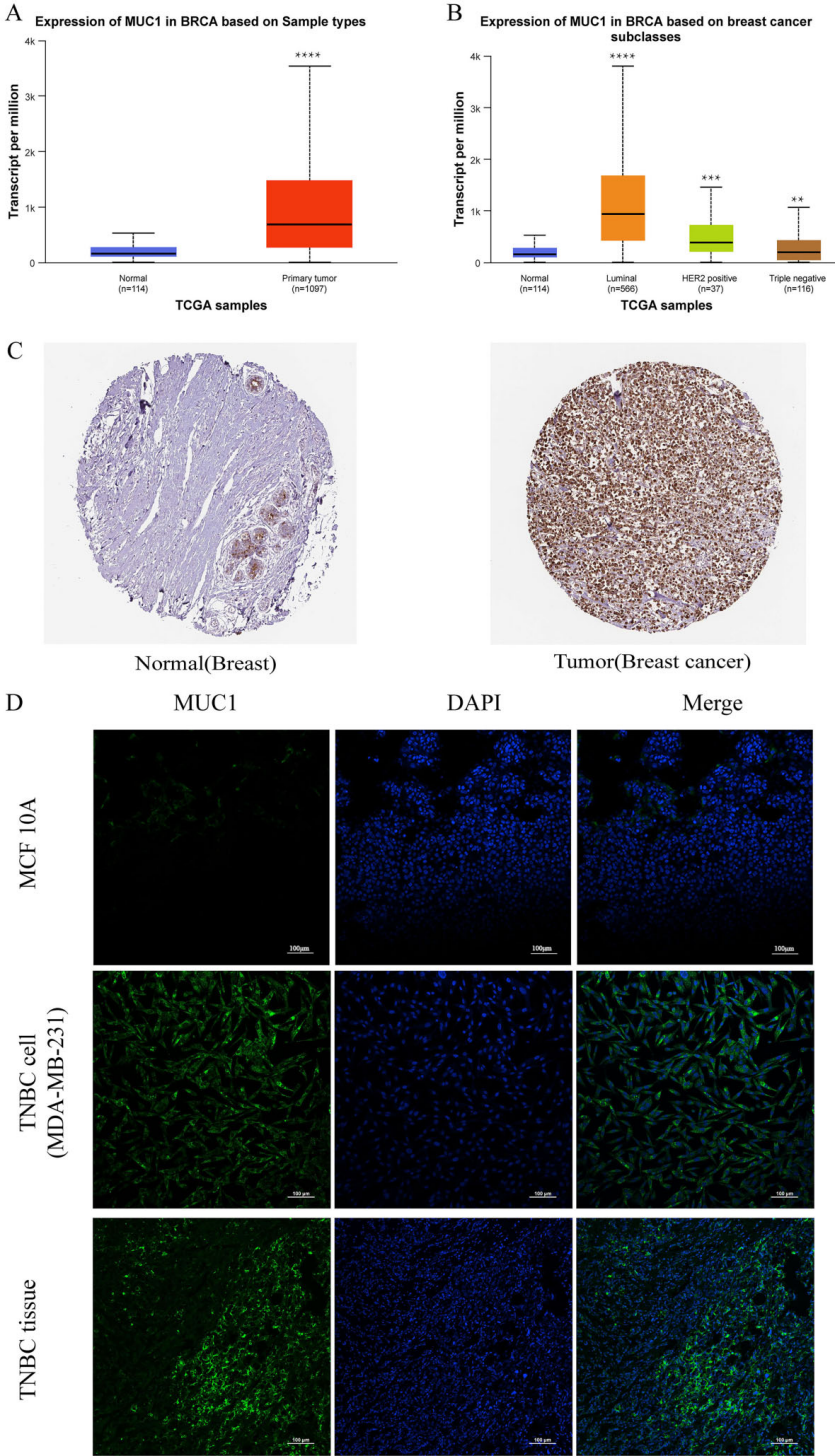
Mucin 1 (MUC1) expression in human breast cancer. **A**, MUC1 expression levels in breast cancer (UALCAN website). **B**, MUC1 expression levels in different subtypes of breast cancer (UALCAN website). **C**, MUC1 protein expression in breast cancer (Human Protein Atlas database). **D**, MUC1 expression in human normal mammary epithelial cells MCF10A, TNBC cells (MDA-MB-231), and TNBC tissues (scale bar=100 μm). Data are reported as means±SE. **P<0.01, ***P<0.001, ****P<0.0001; two-tailed unpaired Student's *t*-test. BRCA: breast cancer gene; TCGA: The Cancer Genome Atlas; TNBC: triple-negative breast cancer.

TCGA is the largest cancer database in the world. We used the UALCAN website (https://ualcan.path.uab.edu/analysis-prot.html) to explore the MUC1 expression level of breast cancer in the TCGA database and to analyze the association of MUC1 expression with cancer subtypes. The results showed that the expression of MUC1 was significantly higher in cancerous breast tissue than adjacent non-tumor breast tissue (P<0.01) (Figure 1A), and it was also significantly higher in luminal, HER2-positive, and triple-negative breast cancer (Figure 1B). The expression of MUC1 protein in breast cancer was explored by immunohistochemical data in the Human Protein Atlas database (https://www.proteinatlas.org/) (Figure 1C). This immunohistochemistry (IHC) staining uses the same antibody staining, but the intensity and quality of the staining are clearly different. Higher expression was detected in breast cancer tissues, and MUC1 expression was mainly restricted to cell cytoplasm and membrane. However, MUC1 protein expression was hardly detected in the adjacent non-tumor breast tissue. In addition, immunofluorescence experiments further confirmed that the oncogene MUC1 was highly expressed in TNBC cells (MDA-MB-231) cells and TNBC tissues. When compared with non-tumor MCF-10A cells, no statistical difference was found (Figure 1D).

### Construction and validation of the MUC1-knockout 231-MUC1-KO cell line

**Figure 2:**
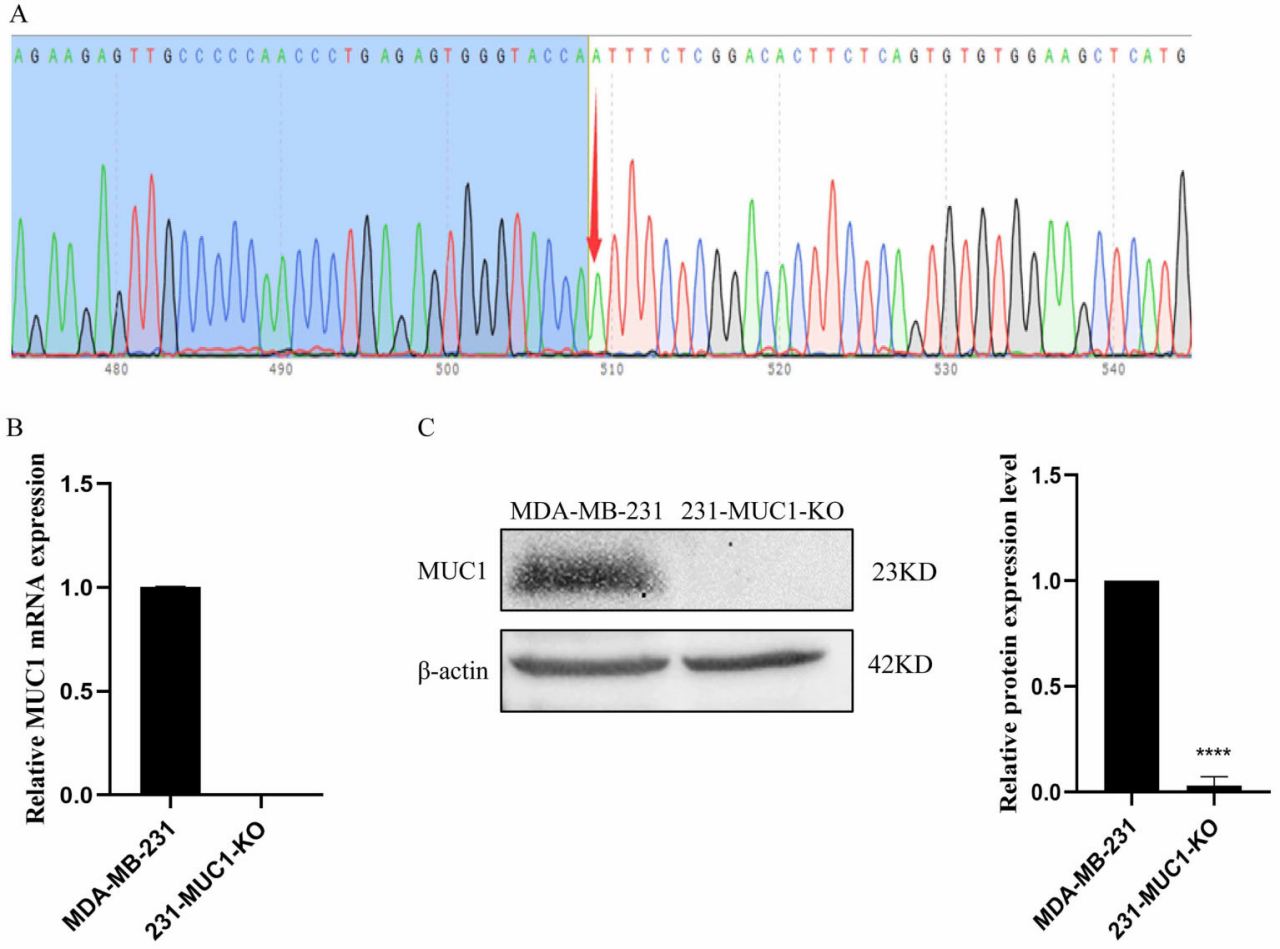
Construction and validation the Mucin 1 (MUC1) knockout cell line 231-MUC1-KO. **A**, Sequencing results after MUC1 knockout were confirmed by the Snapgene software to identify the monoclonal cell line 231-MUC1-KO. **B**, RT-qPCR for MUC1 in MDA-MB-231 and 231-MUC1-KO cells. **C**, Western blot for MUC1 in MDA-MB-231 and 231-MUC1-KO cells. Data are reported as means±SE. ****P<0.0001; two-tailed unpaired Student's *t*-test.

The MDA-MB-231cell line with MUC1 knockout was constructed by the CRISPR/Cas9 technology. The sequencing results were confirmed by Snapgene line 231-MUC1-KO (Figure 2A). RT-qPCR and western blot were used to further confirm the MUC1-KO cell model, and the results showed that the expression of MUC1 mRNA and protein were significantly decreased in the 231-MUC1-KO cell line (Figure 2B).

### MUC1 knockout inhibited proliferation, invasion, and migration of MDA-MB-231 cells

**Figure 3:**
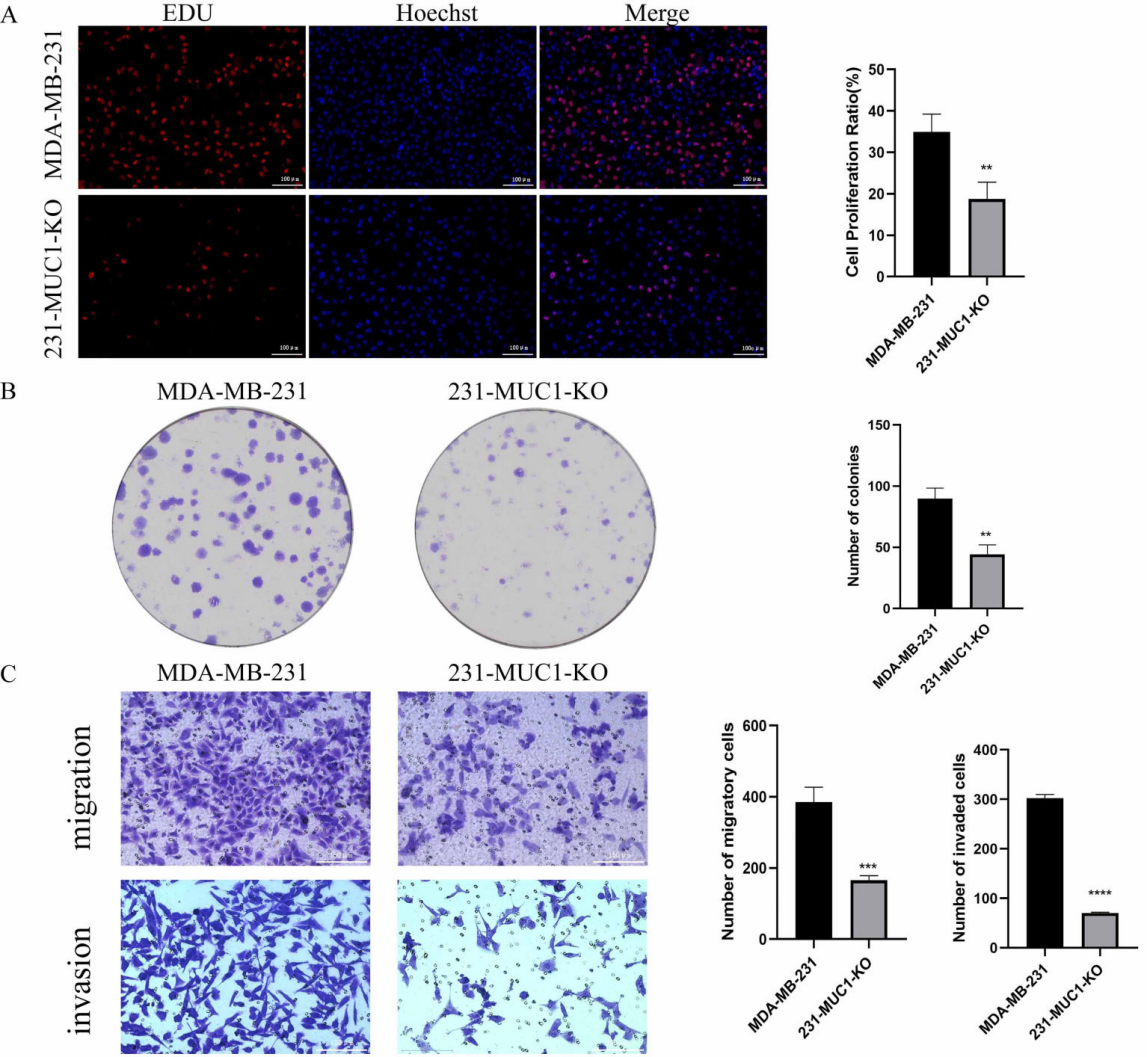
Effect of Mucin 1 (MUC1) knockout on the proliferation, migration, and invasion of MDA-MB-231 cells. **A**, MDA-MB-231 cell proliferation by EDU assay (scale bar=100 μm). **B**, MDA-MB-231 cell proliferation by colony formation assay. **C**, Migration and invasion of MDA-MB-231 cells by transwell assay (scale bar=150 μm). Data are reported as means±SE. **P<0.01, ***P<0.001, ****P<0.0001; two-tailed unpaired Student's *t*-test.

We used the EDU-proliferation assay kit and plate clone formation assay to evaluate the effect of MUC1 knockout on MDA-MB-231 cell proliferation. The results showed that the number of cells with EDU red fluorescent was significantly reduced in the MUC1 knockout group (Figure 3A); the number of cell colonies and cell proliferation capacity in the MUC1 knockout group was significantly lower compared with the control group (Figure 3B). The effect of MUC1 knockout on the migration and invasion ability of MDA-MB-231 cells was assessed using the transwell assay. The results showed that the number of 231-MUC1-KO cells crossing the compartment was significantly reduced compared to MDA-MB-231 cells (Figure 3C).

### Effect of MUC1 knockout on autophagy flow in MDA-MB-231 cells

**Figure 4:**
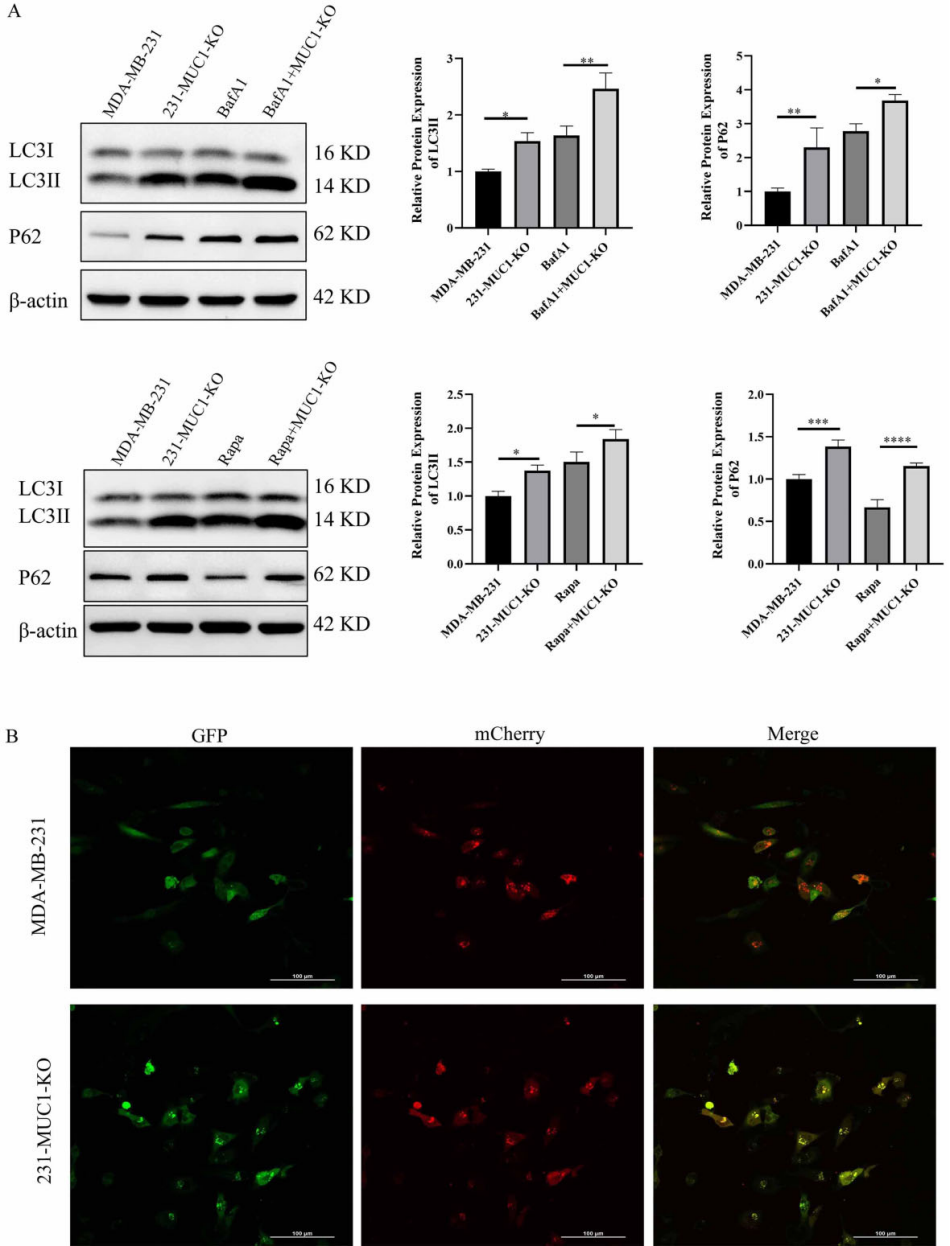
Effect of Mucin 1 (MUC1) knockout on autophagy flow in MDA-MB-231 cells. **A**, Expression of LC3 I, LC3 II, and P62 proteins by western blot. **B**, Ad-mCherry-GFP-LC3B-labeled LC3B protein assessed the patency of intracellular autophagy flow (scale bar=100 μm). Data are reported as means±SE. *P<0.05, **P<0.01, ***P<0.001, ****P<0.0001; two-tailed unpaired Student's *t*-test. BafA1: Bafilomycin A1; Rapa: Rapamycin.

Autophagy flow arrest is known to inhibit tumor cell activity. To further verify whether MUC1 knockout inhibited MDA-MB-231 cell activity by arresting the autophagic flow, we detected the expression of autophagy-related marker proteins (LC3 II, P62) by western blot in 231-MUC1-KO cells. LC3, a putative marker of autophagy, is required for autophagosome formation (6,7). The transition from LC3 I to LC3 II indicates increased autophagosome generation and, on the other hand, may also indicate blocked degradation of autophagosomes. P62 protein is an autophagy substrate involved in the degradation process of autophagy (5,6). It is gradually depleted with the normal progress of autophagy, while the accumulation of P62 reflects autophagy flow damage and is an important marker of autophagy flow blockade. The results showed that the expression of LC3 II and P62 was significantly increased in 231-MUC1-KO cells compared to MDA-MB-231 cells, indicating that MUC1 knockout in MDA-MB-231 cells resulted in autophagic flow arrest (Figure 4A). At the same time, we introduced the autophagy inhibitor bafilomycin A1 as a control and found that after treating cells with it for 24 h, the levels of LC3 II and p62 proteins were significantly upregulated (Figure 4A), indicating that MUC1 knockout and autophagy inhibitor bafilomycin A1 have similar effects on late autophagy. After combined treatment with autophagy inducer rapamycin and MUC1 knockout, the expression of LC3-II and p62 increased significantly compared with treatment with rapamycin alone, indicating that the autophagy flow blockade caused by MUC1 knockout inhibited the degradation of p62 protein induced by rapamycin in cells (Figure 4A).

Ad-mCherry-GFP-LC3B is an adenovirus tool for the detection of autophagy flow (23-25). There is diffuse yellow fluorescence in cytoplasm during non-autophagy, but when an autophagosome is fused with a lysosome, red spots are visible, as the GFP fluorescence is quenched by the acidic environment of the lysosome. We applied the Ad-mCherry-GFP-LC3B two-color fluorescent labeling system to further evaluate the real-time profile of autophagic flow within MDA-MB-231 cells. The results showed a significant increase in autophagosomes (yellow) and a significant decrease in red autophagic lysosomes in 231-MUC1-KO cells compared with the control group (Figure 4B), which further confirmed that the knockout of MUC1 led to the arrest of autophagic flow.

### Effect of MUC1 knockout on lysosomes in MDA-MB-231 cells

**Figure 5:**
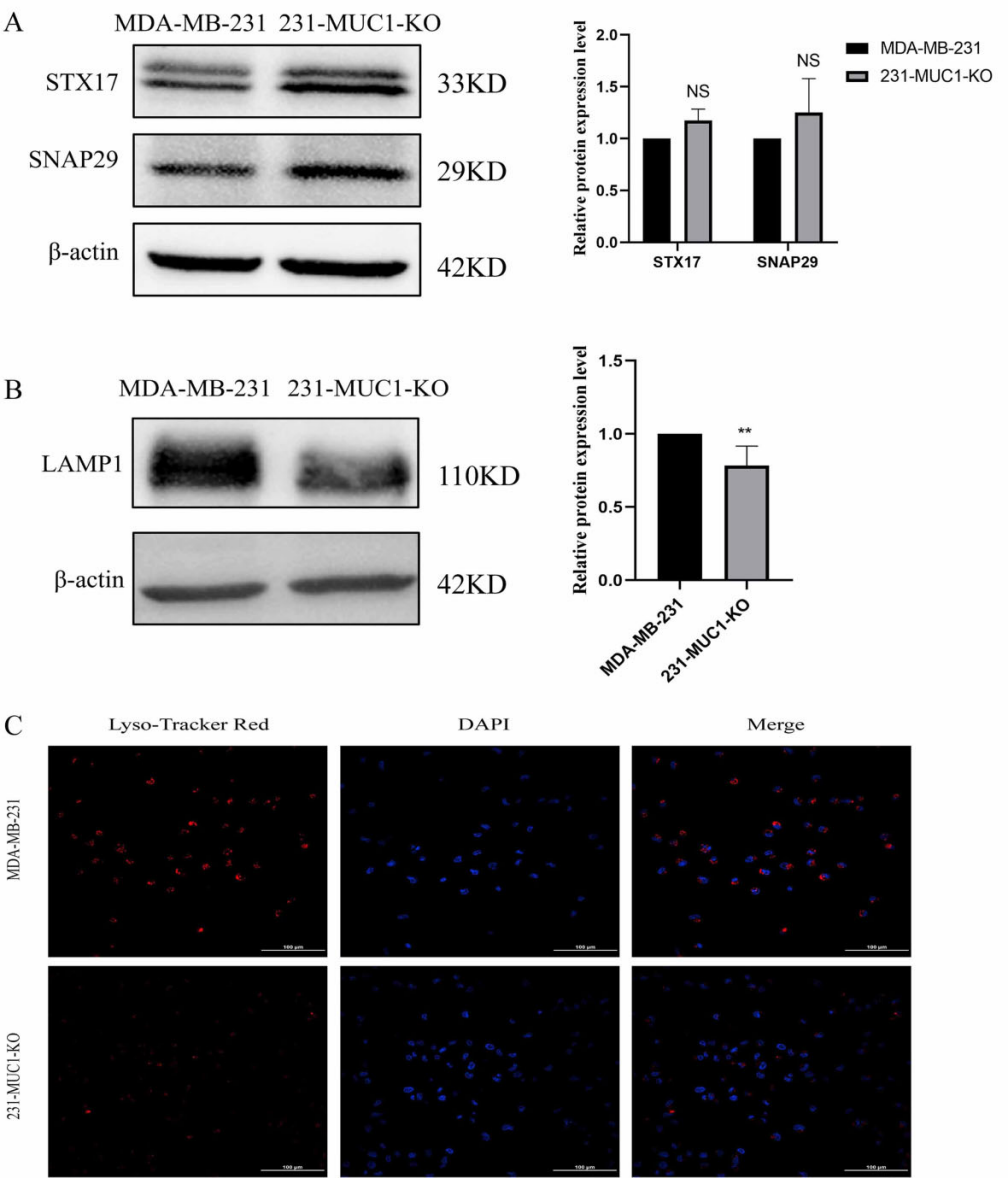
Effect of Mucin 1 (MUC1) knockout on lysosomes in MDA-MB-231 cells. **A**, Expression of SNAP29 and STXl7 by western blot. **B**, Expression of LAMP1 by western blot. **C**, pH was observed with red fluorescent probe Lysotracker Red (scale bar=100 μm). Data are reported as means±SE. **P<0.01, NS: not significant; two-tailed unpaired Student's *t*-test.

We have preliminarily demonstrated that MUC1 knockout affects the activity of MDA-MB-231 cells by inhibiting late autophagy, but the reason for the arrest of autophagic flow is still unknown. During autophagy, the fusion of autophagosome and lysosome is the critical step of autophagy, which is essential for the degradation of autolysosomal contents, and the blocked fusion of both is a common cause for autophagic flow obstruction. We detected the expression level of the key fusion proteins STXl7 and SNAP29 (26,27) by western blot, which were not significantly increased in 231-MUC1-KO cells (Figure 5A), showing that knockout of MUC1 did not block autophagy flow by affecting the fusion between autophagosomes and lysosomes.

Given the above results, combined with the entire process of autophagy, we speculated that the block of autophagic flow may be related to the impairment of lysosomal function. To confirm the hypothesis, the lysosomal membrane landmark protein (LAMP1) (28) and its lysosomal acidification capacity were tested. The results of western blot showed that the expression of LAMP1 protein decreased significantly (Figure 5B), which suggests that the number of lysosomes was decreased. Moreover, the fluorescence expression of Lysotracker Red in 231-MUC1-KO cells was significantly weakened (Figure 5C), which indicates that the pH in lysosomes increased and the degradation ability decreased.

## Discussion

MUC1 is a highly glycosylated transmembrane mucin, mainly expressed in various secretory epithelial cell membranes and at the tip of the secretory pole. It consists of two subunits, MUC1-N and MUC1-C. The MUC1-N subunit contains a variable number of tandem repeats, while the MUC1-C subunit includes an extracellular segment, a transmembrane region, and a cytosolic segment. MUC1-C acts as an oncogene in tumors through oxidative phosphorylation of its cytosolic segment and by mediating intracellular signaling (9-11). Studies have shown that MUC1 can reduce cell adhesion, enhance cell motility, and promote cell invasion and metastasis (10,29,30). It is highly expressed in various malignant tumor tissues, including breast cancer, and is closely associated with tumor stage, lymph node metastasis, vascular invasion, and invasion and metastasis, and is a poor prognostic factor for malignant tumors (31-34). Based on previous studies, we constructed an MUC1 knockout MDA-MB-231 cell model (231-MUC1-KO), which further confirmed that MUC1 knockout can effectively inhibit the proliferation, migration, and invasion of MDA-MB-231 cells *in vitro*, and the potential mechanism may be related to the blockade of autophagic flow caused by lysosomal damage.

We first explored the expression of MUC1 in breast cancer tissue in the TCGA database by the UALCAN website. The results showed that MUC1 expression in human breast cancer tissue was much higher than that of normal breast tissue (Figure 1A), and MUC1 expression in different breast cancer subtypes (luminal, HER2-positive, triple negative) was also higher than that of normal breast tissue. However, another online data analysis showed increased MUC1 expression in various pathological types (34). When exploring the relationship with molecular subtypes, MUC1 expression was significantly increased in ER+ or PR+ cases, but significantly downregulated in TNBC, and no difference in expression in HER+/- patients (34). Quantitative PCR data (35) showed that MUC1 expression in breast cancer was 15.1 times higher than normal breast tissue and was independent of ER, PR, or HER2 type. Data (36) from IHC of breast cancer tissue showed that MUC1 was expressed in over 90% of the entire breast cancer population and significantly increased in ER+ cases (PR+, HER2-positive, and triple negative were not included in the study) compared with 94% in TNBC cases (37) (82.5% reported by Abtin et al. (35)). Atta Manu et al. (33) recorded 59% MUC1-positive cases in a study in Ghana, in which MUC1 overexpression was significantly association with the HER2 overexpression phenotype and the triple-negative subtype. IHC data in the Human Protein Atlas database showed increased MUC1 protein expression in breast cancer tissue, but rarely expressed in normal breast tissue. Therefore, we showed that MUC1 is widely and highly expressed in breast cancer tissue, and differences in subgroup analysis according to molecular typing may be related to geographical region, sample size, detection method, among other factors. We similarly confirmed that MUC1 expression was significantly increased in MDA-MB-231 cells and TNBC tissues by immunofluorescence experiments. Therefore, the widespread high expression of MUC1 in breast cancer tissues further proves its universality as a new target for breast cancer and even TNBC therapy. Currently, a large number of targeted therapies targeting MUC1 in TNBC have been carried out.

MUC1 is highly expressed in TNBC tissues, and its overexpression is associated with the invasiveness and metastatic properties in various solid tumors including breast cancer (31-33,38). Studies (10,29,30,39) have proven that MUC1-C can induce epithelial-to-stromal transformation through its interaction with pro-inflammatory transcription factors (for example, NF-κB and STAT3) in the nucleus to initiate the expression of downstream target genes (ZEB1, TWIST1, and SNAIL, etc.), making tumor cells more aggressive and prone to metastasis. Interference of circular RNA circ_0009910 downregulated the expression of MUC1 by miR-145-5p, which significantly reduced the proliferation and migration capacity of breast cancer MCF-7 cells (35). However, direct interference with MUC1 gene expression inhibited pancreatic cancer cell migration and metastasis through TGF-β/AMPK/Sirt1/FGF21 signaling pathway. Our study similarly found that MUC1 knockout effectively inhibited cell proliferation, migration, and invasion compared with MDA-MB-231 cells, which further demonstrated its role as an oncogene in tumor development.

Li et al. (40) reported that inhibition of MUC1 could mediate mitophagy through the ATAD3A/Pink1 axis and significantly inhibit MUC1-positive cancer cells. Our study similarly showed a significant increase in the expression of LC3 II and P62 protein in 231-MUC1-KO cells, indicating that the MUC1 knockout led to an accumulation of autophagosome and autophagic substrate, which may be associated with increased autophagosome generation or be related to the blocked autophagosome degradation process. In addition, we also detected the changes of autophagy flow by the Ad-mCherry-GFP-LC3B double labeled plasmid, indicating that after MUC1 knockout, the vast majority of autophagosomes in the cell exhibited aggregated, yellow (a fusion color of red and green fluorescence) dot-like structures distributed within the cell. This indicates that autophagosomes generated within the cell cannot be wrapped or engulfed by lysosomes with acidic internal environment, and thus complete the corresponding late degradation process (autophagosomes that can be wrapped by acidic lysosomes will exhibit yellow fluorescence, as green fluorescence is easily quenched under acidic conditions). Therefore, we confirmed that the MUC1 knockout can have an inhibitory effect on autophagy flow (that is, autophagy flow arrest), which can effectively inhibit the proliferation, migration, and invasion of MDA-MB-231 cells, but the specific mechanism of autophagy flow arrest is still unclear.

Autophagy flow arrest can also occur at later stages. In the late stage of autophagy, autophagosomes fuse with lysosomes to completely enclose the material to be digested before degradation and recycling. Autophagosome-localized STXl7 and SNAP29 and lysosomal-localized VAMP8 and VAMP7 have been suggested to act as key factors involved in the fusion process (26). After the initiation of autophagy, STXl7 translocates to the autophagosome and forms a binary complex with SNAP29, and then STXl7-SNAP29 interacts with lysosomal VAMP8 to form a membrane complex that promotes the autophagosome-lysosome fusion (27). Therefore, we explored the ability of lysosomal generation, lysosome fusion, and lysosomal acidification capacity in order to investigate the reason of the autophagy flow arrest. We found that MUC1 knockout did not significantly affect the expression of STXl7 and SNAP29 proteins, suggesting that MUC1 knockout induces autophagy, but does not hinder autophagy flow by affecting the fusion of autophagosomes and lysosomes. Lysosomal landmark protein LAMP1 plays an important role in maintaining the structural stability of lysosomal membrane, and the decrease in its expression will affect autophagy function (28). In this study, LAMP1 protein expression decreased after knockout of MUC1, suggesting a decrease in the number of lysosomes. The Lysotracker Red probe can be selectively retained in more acidic lysosomes, thus achieving specific fluorescent labeling for lysosomes, allowing the detection of pH changes within lysosomes. The Lysotracker red fluorescence intensity indicated the increase of pH and the alkalization of lysosomes.

## Conclusions

**Figure 6:**
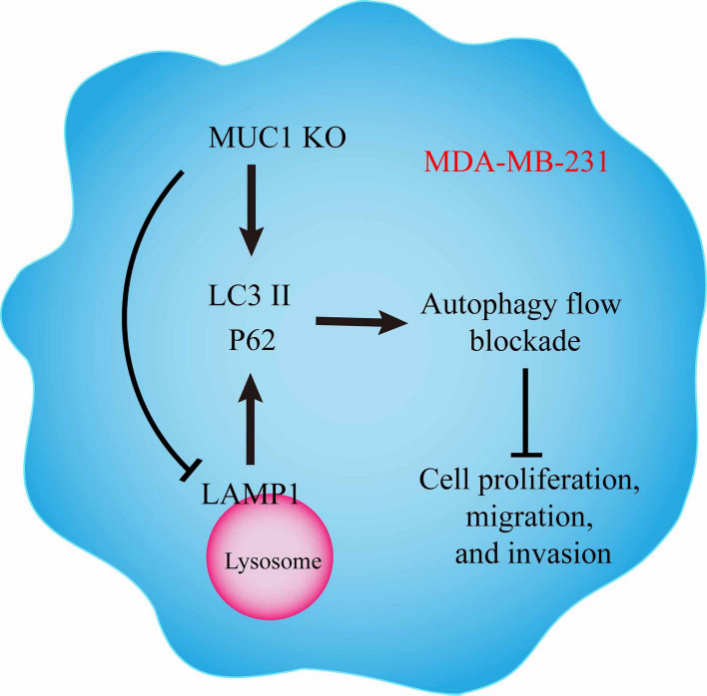
Schematic diagram of the Mucin 1 (MUC1) oncogenic mechanism in breast cancer cell line MDA-MB-231. Knockout (KO) of MUC1 inhibits the proliferation, migration, and invasion of MDA-MB-231 cells by regulating the autophagic flow, and the possible mechanism may be related to the blocking of the autophagic flow caused by lysosomal damage.

The above findings indicate that knocking out MUC1 causes damage to the lysosomes of MDA-MB-231 cells, leading to a decrease in lysosome count and pH alkalization, and autophagic flow arrest (Figure 6). Autophagosomes cannot be degraded normally and accumulate in the cells, resulting in an increased number of autophagosomes, ultimately inhibiting MDA-MB-231 cell proliferation, migration, and invasion. However, this study had some limitations such as using only one breast cancer cell line, the lack of further validation with autophagy inducers, and the absence of animal experiments. We will next explore the relationship between autophagy and tumor immunity.

## Data Availability

The entire dataset supporting the results of this study is published in the article itself.
